# The Role of Cytokines in Vascular Endothelial Glycocalyx Integrity and Impairment Following Open-Heart Surgery

**DOI:** 10.3390/biomedicines14040837

**Published:** 2026-04-07

**Authors:** Lara Batičić, Božena Ćurko-Cofek, Gordana Taleska Štupica, Matej Jenko, Marko Zdravković, Lea Cofek, Antea Krsek, Tanja Batinac, Danijel Knežević, Marino Damić, Mia Šestan, Aleksandra Ljubačev, Maja Šoštarič, Vlatka Sotošek

**Affiliations:** 1 Department of Medical Chemistry, Biochemistry and Clinical Chemistry, Faculty of Medicine, University of Rijeka, Braće Branchetta 20, 51000 Rijeka, Croatia; lara.baticic@uniri.hr; 2 Department of Physiology, Immunology and Pathophysiology, Faculty of Medicine, University of Rijeka, Braće Branchetta 20, 51000 Rijeka, Croatia; 3 Clinical Department of Anaesthesiology and Surgical Intensive Care, University Medical Centre, Zaloska 7, 1000 Ljubljana, Slovenia; taleskagordana@gmail.com (G.T.Š.); matej.jenko@kclj.si (M.J.); maja.sostaric@kclj.si (M.Š.); 4 Medical Faculty, University of Ljubljana, Vrazov trg 2, 1000 Ljubljana, Slovenia; 5 Department of Anaesthesiology, Intensive Care and Pain Management, University Medical Centre Maribor, Ljubljanska ulica 5, 2000 Maribor, Slovenia; markozdravkovic@gmail.com; 6 Faculty of Medicine, University of Rijeka, Braće Branchetta 20, 51000 Rijeka, Croatia; lea.cofek@gmail.com (L.C.); tea.krsek@gmail.com (A.K.); 7 Department of Clinical Medical Sciences I and II, Faculty of Health Studies, University of Rijeka, Viktora Cara Emina 2, 51000 Rijeka, Croatia; tanja.batinac@uniri.hr (T.B.); vlatkast@uniri.hr (V.S.); 8 Department of Anesthesiology, Reanimatology, Emergency and Intensive Care Medicine, University of Rijeka, Braće Branchetta 20, 51000 Rijeka, Croatia; danijel.knezevic2@uniri.hr; 9 Clinic of Anesthesiology, Intensive Medicine and Pain Management, Clinical Hospital Center Rijeka, Krešimirova 42, 51000 Rijeka, Croatia; damic1@live.com (M.D.); mia.sestan1@gmail.com (M.Š.); 10 Department of Surgery, Faculty of Medicine, University of Rijeka, Braće Branchetta 20, 51000 Rijeka, Croatia; aleksandra.ljubacev@uniri.hr

**Keywords:** cytokines, endothelial glycocalyx, inflammation, open-heart surgery, oxidative stress, systemic inflammatory response, vascular endothelium

## Abstract

Open-heart surgery with cardiopulmonary bypass (CPB) is a high-risk procedure with significant morbidity and mortality. CPB, tissue injury, blood loss, endotoxemia and ischemia–reperfusion injury induce a pronounced systemic inflammatory response, leading to endothelial glycocalyx (EG) damage and vascular endothelial dysfunction. Consequently, immune cells, reactive oxygen species, and enzymes gain free access to vascular endothelial cells, resulting in their dysfunction and enhancing inflammation, vascular permeability, and microvascular impairment. EG degradation is most commonly assessed by measuring the circulating levels of its degradation products. Additionally, CPB triggers an early inflammatory response that is characterized by the secretion of interleukin (IL)-1β, IL-6, IL-8, tumor necrosis factor alpha, and IL-18, which play roles in initiating the process of EG injury. EG damage is further propagated by the sustained release of cytokines, inhibiting the regeneration of the glycocalyx layer. Heparanase and matrix metalloproteinases are enzymatic pathways involved in cytokine-mediated EG degradation after cardiac surgery, and the balance between the pro- and anti-inflammatory cytokines determines the magnitude and duration of the inflammatory response and EG impairment, which correlates with adverse clinical outcomes, including myocardial dysfunction, acute lung and kidney injury, neurological complications, and prolonged need for intensive care. Thus, identifying patients with an exaggerated cytokine response could potentially provide more personalized therapy based on the circulating biomarkers of EG shedding, and cytokine-directed preservation of EG represents a promising therapeutic strategy in vascular dysfunction prevention during and after open-heart surgery. In this review, we summarize the current knowledge on cytokine-mediated EG impairment following open-heart surgery with CPB.

## 1. Introduction

Despite continuous innovations in surgical techniques, cardiac surgery remains a high-risk procedure associated with adverse events, perioperative complications, and hospital mortality [1]. It is believed that inflammation plays a significant role in most of these negative processes and outcomes [2]. Cardiopulmonary bypass (CPB), which is used in cardiac surgery, enables complex cardiac surgical procedures by replacing heart and lung function during surgery, thereby allowing a bloodless surgical field and maintaining systemic perfusion [3]. However, despite its advantages, CPB greatly contributes to the development of a systemic inflammatory response [4].

Non-pulsatile CPB is the most commonly used variant. However, in an attempt to mimic the physiological arterial pulse, which may be more protective for organs, pulsatile CPB has been developed [5]. Nevertheless, contact between blood and the artificial surfaces of the CPB circuit initiates a cascade of adverse events, such as complement and leukocyte activation and endothelial cell damage. The effects of CPB, combined with those of the surgical procedure itself, such as tissue injury, blood loss, and ischemia–reperfusion injury, result in the release of pro-inflammatory cytokines, the development of oxidative stress, and an amplified inflammatory response [2,6].

Pro-inflammatory cytokines contribute to vascular endothelial dysfunction and the loss of the protective barrier provided by the endothelial glycocalyx (EG) that covers the surface of endothelial cells. Consequently, immune cells, together with other deleterious agents such as reactive oxygen species (ROS) and enzymes, gain free access to vascular endothelial cells, resulting in their dysfunction [7].

The aim of this narrative review was to analyze the complex interplay between cytokine signaling and endothelial integrity in patients undergoing open-heart surgery.

## 2. Methods: Search Strategy

This narrative review was conducted to summarize and critically evaluate the current evidence regarding the role of cytokines in mediating the integrity and impairment of the vascular EG following open-heart surgery. The following keywords were used: cytokines; endothelial glycocalyx; inflammation; open-heart surgery; oxidative stress; systemic inflammatory response; and vascular endothelium.

The PubMed and Web of Science databases were searched to identify relevant studies published up to February 2026, with an emphasis on recent, high-quality publications. Additional relevant articles were identified by manual screening reference lists.

Eligible studies included original research articles, longitudinal cohort studies, and relevant review articles, while non-English-language studies, case reports, and those not directly relevant to this study were excluded.

The selected studies were synthesized narratively to identify key themes, areas of consistency, and gaps in the current body of knowledge. As this is a narrative review, no formal risk-of-bias assessment or quantitative synthesis was performed, and we acknowledge this fact as a limitation of the current study.

## 3. EG Structure and Function

In general, the EG is a gel-like coating comprising glycosylated lipid–protein complexes [8]. It covers the surface of all living cells, creating a protective layer between the cells and the blood [9].

Vascular endothelial cells synthesize and secrete EG on their luminal surface throughout the vascular system, from capillaries to large arteries and veins [10,11,12]. It provides a semipermeable interface and protects endothelial cells from harmful agents such as cytokines and oxidants [13]. Additionally, the EG varies in thickness and composition depending on the functional demands of each organ [11,14]. For instance, in continuous capillaries, it is thicker and more tightly organized [15]. In contrast, fenestrated capillaries contain a thinner, more selectively permeable EG, thereby allowing easier transport of molecules [14].

**Figure 1:**
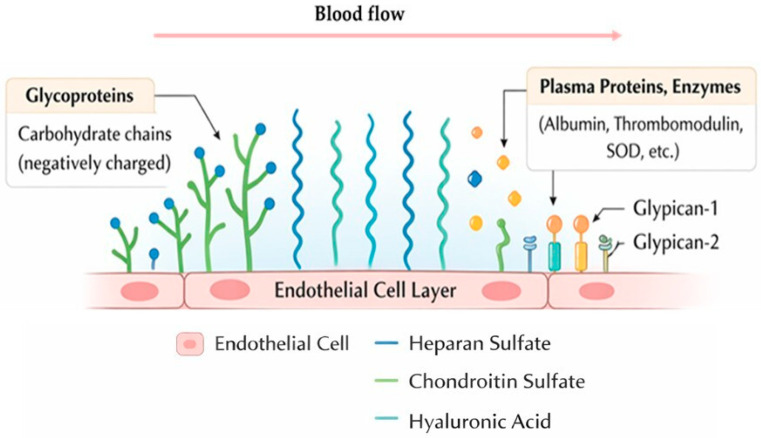
Schematic illustration of the endothelial glycocalyx (EG) structure. The EG covers the luminal side of the vascular endothelial cells. It is composed of a mixture of glycoproteins with carbohydrate chains, glycosaminoglycans (such as heparan sulfate, chondroitin sulfate, and hyaluronic acid), and proteoglycans (like glypican-1 and glypican-2), and different plasma proteins (albumin, thrombomodulin, superoxide dismutase (SOD), etc.) are found close to the endothelial cell layer.

The EG is composed of a mixture of proteoglycans, glycoproteins with acidic oligosaccharides and terminal sialic acid, glycosaminoglycans (GAGs), and glycosphingolipids [14,16], and some of these components are anchored to the cell membrane by transmembrane domains or covalent bonds, while others are indirectly attached to the cell via receptor molecules [17], as shown on Figure 1.

Glycoproteins and proteoglycans provide structural support to the glycocalyx [11,18], and the most common proteoglycans in the EG are syndecans and glypicans. Syndecans (syndecan-1 to -4) are transmembrane proteins with cytoplasmic tails that undergo oligomerization and interact with protein kinase C [17,19]. In contrast, glypicans (glypican-1 to -6) attach to the cell membrane using glycosylphosphatidylinositol molecules [17]. Other endothelial proteoglycans (e.g., mimecan and perlecan) are soluble and present in both the EG and the blood [19].

Glycoproteins consist of short carbohydrate chains capped with sialic acid sugar residues [20], and many EG glycoproteins are cell adhesion molecules belonging to the selectin, immunoglobulin, or integrin family. They have small cytoplasmic tails, transmembrane sections, and variable extracellular domains, and these extracellular domains confer specific functional properties to glycoproteins. Additionally, intercellular adhesion molecule (ICAM), platelet endothelial cell adhesion molecule (PECAM), and vascular cell adhesion molecule (VCAM) are all glycoprotein cell adhesion molecules of the EG [17].

GAGs are linear, anionic polysaccharides composed of uronic acids and hexosamine residues that undergo acetylation, sulfation, and epimerization [21]. They are continuously degraded by enzymes, but their concentration is maintained by the Golgi apparatus, which regulates their constant synthesis [22]. The five main components of GAG side chains are heparan sulphate, chondroitin sulphate, dermatan sulphate, keratan sulphate, and hyaluronan, with heparan sulphate being the most abundant [11]. Heparan sulphate chains interact with various growth factors, cytokines, and enzymes, thereby regulating cell proliferation, angiogenesis, and inflammation. In addition, they serve as binding sites for anticoagulant molecules and thus participate in the inhibition of the coagulation cascade [16]. Hyaluronan is the only non-sulfated GAG chain that does not covalently bind to proteins [19]. It is anchored to the cell via the cell membrane receptor CD44 [17]. Hyaluronan possesses strong polymerization capabilities and is incorporated deep within the EG, closer to the endothelial cells [19]. It assists in hydrating and lubricating the EG, thereby enhancing its resilience and extending its anti-adhesive properties. Glycosphingolipids are lipid molecules containing carbohydrate moieties, and they associate with lipid raft domains and regulate cell-surface dynamics and receptor function [16]. In addition to the major structural components of the EG, it also contains both soluble and insoluble molecules, which include plasma proteins, enzymes, cofactors, and enzyme inhibitors, such as albumin, superoxide dismutase, xanthine oxidoreductase, thrombomodulin, and antithrombin III [19,23]. Together, these components contribute to EG homeostasis maintenance [17].

EG plays a crucial role in maintaining vascular homeostasis and regulating numerous physiological processes [24,25]. It serves as the primary interface between circulating blood and the vascular wall, actively participating in mechanotransduction, vascular permeability, and the modulation of inflammatory responses [26,27]. Mechanotransduction refers to the mechanical-to-biochemical conversion of signals [28]. In the vasculature, the EG translates blood shear forces to functional and genetic changes inside the endothelial cells, and heparan sulfate has been found to act as a primary sensor in this process. Additionally, the mechanotransduction process is pivotal for triggering nitric oxide (NO) release through the activation of endothelial nitric oxide synthase (eNOS) on the endothelial surface [29]. NO regulates vasomotor tone and peripheral oxygen distribution [30].

Beyond its role in vasoregulation, the EG controls the extravasation of fluids, ions, and molecules. Its strategic location enables it to mediate flow-induced shear stress on endothelial cells by acting as a selective permeable barrier that prevents transvascular protein leakage and reduces leukocyte–endothelial interactions [26,31]. The semi-permeable nature of the EG allows the passage of certain macromolecules like plasma proteins while impeding larger entities such as red blood cells or dextrans [32]. Moreover, the EG regulates the access of circulating cells and molecules to the endothelium [24], thereby modulating thrombocyte adhesion and leukocyte tissue recruitment [33].

It also actively participates in antithrombotic processes by binding antithrombin and enhancing its inhibitory effect on thrombin, thereby preventing excessive coagulation [7]. Its constituents, such as thrombomodulin, a tissue factor pathway inhibitor, as well as heparan and dermatan sulfate, are integral to its anticoagulant and anti-thrombotic properties [29]. Furthermore, the EG also influences von Willebrand factor activity, potentially regulating its adhesion to the endothelium and subsequent involvement in coagulation cascades, particularly under inflammatory conditions where its integrity may be compromised [34]. Disruption or shedding of the EG exposes endothelial cell surface adhesion molecules, including selectins and integrins, facilitating neutrophil rolling, firm adhesion, and trans-endothelial migration. Concurrently, EG degradation enhances neutrophil activation and promotes the release of neutrophil extracellular traps (NETs), which provide a structural scaffold for platelet adhesion and fibrin deposition, thereby amplifying thrombus formation [35]. Loss of EG integrity also exposes the subendothelial matrix and reduces the spatial barrier between circulating platelets and von Willebrand factor, leading to increased platelet adhesion, activation, and aggregation. In parallel, impaired EG function is associated with reduced bioavailability of NO and other anticoagulant mediators, further shifting the endothelial surface toward a procoagulant state. These changes establish an environment for immunothrombosis that, together with enhanced platelet–subendothelial interaction, synergistically contributes to microvascular and macrovascular thrombosis [12].

Apart from the aforementioned functions, the EG also plays a role in mediating leukocyte adhesion [36,37]. Leukocyte activation during inflammatory and immune responses is critically regulated by the EG, which acts as a protective barrier, shielding the endothelium from excessive interactions with circulating leukocytes [7]. Specifically, interactions between the leukocytes and endothelial cells are mediated by adhesion molecules (selectins and integrins). An intact glycocalyx conceals these adhesion molecules, thereby minimizing leukocyte–endothelial interactions [29]. However, under pathological conditions, EG degradation compromises this protective function, directly contributing to increased endothelial permeability and facilitating immune cell access to the vessel wall [7].

## 4. The Role of the EG During and Following Open-Heart Surgery

Open-heart surgery induces endothelial injury through multiple interrelated mechanisms, including surgical trauma, CPB, ischemia–reperfusion injury, inflammatory activation, oxidative stress, and blood exposure to artificial surfaces. Accordingly, cardiac surgery is recognized as a potent trigger of systemic inflammation and oxidative stress, both of which directly damage endothelial cells and promote EG degradation. CPB-driven cytokine release, ROS generation, and complement activation collectively contribute to the enzymatic shedding of EG components [38]. Endothelial dysfunction is considered a central pathophysiological feature of cardiac surgery, with EG degradation representing an early manifestation of extracorporeal circulation-induced injury. In this context, inflammatory mediators, oxidative stress, and protease activation act synergistically to disrupt the endothelial surface layer [39]. Moreover, evidence linked CPB duration to EG injury, demonstrating that prolonged extracorporeal circulation increases the release of soluble EG components and damage-associated molecular patterns, reflecting endothelial stress [40].

The severity of EG degradation varies according to the surgical approach. On-pump coronary artery bypass graft (CABG) results in the most pronounced shedding, whereas off-pump CABG reduces, but does not eliminate, EG injury, as localized ischemia and oxidative stress still persist [38]. EG degradation contributes to microvascular dysfunction and is associated with adverse clinical implications, leading to postoperative complications that prolong the course of patients’ recovery [40,41,42].

The EG is a dynamic structure. Reitsma et al. described it as a self-assembling endothelial surface layer in which enzymatic removal of individual components compromises barrier function and mechanotransduction, underscoring the interdependence of its structural elements [41].

Among proteoglycans, syndecan-1 is the most extensively studied marker of EG degradation. Robich et al. demonstrated significant postoperative increases in circulating syndecan-1 following CPB, with its levels proportional to CPB duration. Peak levels were observed several hours after CPB cessation and remain elevated for up to 24 h postoperatively, indicating sustained EG disruption [40]. Furthermore, Li et al. identified glypican-1 shedding as an additional marker of endothelial dysfunction in prolonged CPB (>180 min), with elevated postoperative levels correlating with injury severity. Additionally, increased levels of matrix metalloproteinase (MMP)-9 and interleukin (IL)-1β indicated ongoing endothelial and inflammatory activation [42]. Clinical studies have delineated the timing of EG degradation during cardiac surgery. Bol et al. used a multimodal assessment during CABG to demonstrate that EG thinning occurs immediately after CPB initiation, as evidenced by an increased perfused boundary region (PBR) and elevated circulating levels of syndecan-1, heparan sulfate, and hyaluronan. Notably, these changes plateau during CPB, suggesting that initiation represents the critical time point of injury [43].

GAGs also play a critical role in EG integrity. Henry showed that hyaluronan regulates permeability, as its enzymatic degradation selectively increases macromolecular penetration into the endothelial surface layer without altering vessel diameter or red blood cell exclusion [44]. Aldecoa et al. further emphasized the stabilizing role of plasma proteins, particularly albumin, noting that the loss of EG components disrupts albumin–endothelium interactions and increases vascular permeability, leading to postoperative complications [45].

Kim et al. found that elevated preoperative syndecan-1 expression independently predicted severe acute kidney injury after valvular surgery and was associated with systemic inflammation, increased right ventricular systolic pressure, and prolonged hospitalization [46]. Moreover, Robich et al. linked syndecan-1 levels to postoperative neutrophil counts, suggesting a connection between EG shedding and inflammatory cell activation [40]. Patterson et al. further contextualized these findings by framing EG degradation as a common pathway of endothelial injury in critical illness, identifying syndecan-1 and heparan sulfate as clinically relevant biomarkers [7]. Thus, a clinical study reported that increased levels of syndecan-1 early after pediatric cardiac surgery are associated with severe acute kidney injury [47]. Since acute kidney injury develops in nearly 40% of cardiac surgery patients and leads to mortality rates up to 80%, identifying a reliable early biomarker is of great importance. Xu et al. reported that elevated syndecan-1 levels are associated with fluid overload and progressive acute kidney injury after cardiac surgery [48], and a study by Budiwardhana et al. showed that syndecan-1 kinetics reflect adverse postoperative outcomes, including low cardiac output syndrome, in children undergoing cardiac surgery [49]. Therefore, it is important to quantitatively assess EG degradation markers using appropriate methodological approaches.

Recently, both sidestream dark-field and incident dark-field (SDF/IDF) imaging have gained attention as techniques for evaluating EG integrity [50]. These methods rely on the optical detection of flowing erythrocytes within the microvasculature. An intact EG excludes erythrocytes from the endothelial surface, and alterations in their lateral penetration can be quantified using the PBR [51]. An increased PBR reflects deeper erythrocyte penetration into the glycocalyx layer and is therefore interpreted as a surrogate marker of EG thinning or degradation [50]. Complementary to imaging-based approaches, circulating biomarkers released during glycocalyx shedding provide biochemical evidence of EG degradation.

## 5. The Effects of Cytokines on the EG During Open-Heart Surgery

Open-heart surgery with CPB triggers a profound systemic inflammatory response that is characterized by the release of multiple cytokines, including pro-inflammatory mediators such as IL-1β, IL-6, IL-8, IL-18, and tumor necrosis factor-alpha (TNF-α), as well as anti-inflammatory cytokines like IL-10 [52,53]. These cytokines play crucial roles in orchestrating the immune response, regulating vascular permeability, modulating coagulation pathways, and influencing end-organ function [54]. Thus, understanding the temporal dynamics and clinical implications of cytokine release during open-heart surgery is essential for anesthesiologists and intensivists managing these complex patients. Cytokine profiling may serve as a valuable tool for risk stratification, early identification of patients at high risk for complications, and targeted therapeutic intervention guidance [55] since traditional inflammatory biomarkers such as leukocyte count, C-reactive protein (CRP), and procalcitonin have their limitations in specificity and timing [56].

CPB induces a complex systemic inflammatory response through multiple mechanisms [52,54], but the three principal triggers are (1) blood contact with artificial surfaces of the CPB circuit, thereby activating the complement, coagulation, and contact activation systems; (2) ischemia–reperfusion injury to the heart, lungs, and other organs; and (3) endotoxemia resulting from splanchnic hypoperfusion and gut barrier dysfunction [52,53,54]. These triggers initiate a cytokine cascade that begins intraoperatively and continues into the postoperative period. Pro-inflammatory cytokines (IL-6, IL-8, and TNF-α) are released early, followed by compensatory anti-inflammatory mediators, including IL-10 [53,57]. The magnitude and duration of this inflammatory response correlate with adverse clinical outcomes, including myocardial dysfunction, acute lung and kidney injuries, neurological complications, and prolonged intensive care unit stays [55,58]. The balance between pro-inflammatory and anti-inflammatory cytokines determines the clinical trajectory of patients undergoing cardiac surgery, as excessive pro-inflammatory responses can lead to vasoplegic syndrome, capillary leakage, and multi-organ dysfunction, while inadequate inflammatory control may result in immunosuppression and increased infection risk [54].

IL-6 emerged as the most consistently and robustly elevated cytokine during open-heart surgery with CPB [52,53,55]. Multiple studies documented significant IL-6 elevation beginning during CPB and peaking 2–6 h postoperatively [53,55,58]. Peak IL-6 levels were strongly correlated with CPB duration, with longer bypass times associated with higher cytokine concentrations [58]. Halter et al. demonstrated systematic IL-6 release in patients undergoing CPB, with levels correlating with postoperative lung dysfunction [53]. Holmes et al. identified “hyper-responders” with exaggerated IL-6 and IL-8 levels that peak at approximately 4 h post-CPB who experienced higher rates of delayed extubation, bleeding complications, and worse early functional outcomes [55]. Habes et al. confirmed that peak IL-6 expression occurred around 4 h post-CPB and that an integrated cytokine Z-score (including IL-6) correlated with postoperative troponin elevation and greater postoperative organ dysfunction [58]. Martínez-Comendador et al. showed that preoperative statin treatment reduced both postoperative IL-6 levels and troponin/CK-MB elevations, supporting the linkage between cytokine responses and myocardial injury while demonstrating the modifiability of this relationship [59].

IL-6 is known to play a distinct role in EG impairment. Unlike the other cytokines, such as TNF-α and IL-1β, that are responsible for the structural injury to the EG, IL-6 contributes to the persistence of endothelial dysfunction by maintaining chronic inflammatory signaling [60,61].

Prolonged expression of IL-6 has correlated with delayed recovery of EG thickness and endothelial repair mechanisms, and IL-6 signaling has been demonstrated to interfere with endothelial homeostasis, including proteoglycan synthesis and NO signaling, thereby impairing endothelial regenerative capacity. Thus, IL-6 is now considered to play a role in the pathophysiology of subacute rather than acute EG shedding [62]. It also plays a role in activating endothelial cells by maintaining the inflammatory response and extending ROS production in the postoperative period [63,64,65].

IL-8 demonstrated particularly strong associations with hemodynamic instability and respiratory complications. In a prospective pediatric study, Saelim et al. reported that an increase in IL-8 (>56 pg/mL) from baseline to immediate postoperative measurement was strongly associated with low cardiac output syndrome with an odds ratio of 37.34 [66]. This finding highlights the potential utility of IL-8 as an early biomarker for hemodynamic compromise. It is known that cytokine-mediated vasodilation represents a significant clinical challenge in cardiac surgery [67,68]. Wei et al. found that higher IL-6 and IL-8 levels were observed in patients requiring vasopressors in the intensive care unit, with IL-8 levels correlating with norepinephrine dosage requirements [67]. Multiple studies documented associations between elevated IL-8 levels and prolonged mechanical ventilation [55,67]. Additionally, an increased concentration of IL-8 following CPB promotes its binding to GAGs on endothelial cell surfaces, inducing neutrophil recruitment and thereby amplifying the proinflammatory response [69].

Other early cytokines include IL-18 and its antagonist IL-18 binding protein (IL18-BP). Due to the expression of IL-18 in endothelial cells, smooth muscle cells of blood vessels, cardiomyocytes, and macrophages, it has pleiotropic effects on immune cells and the vascular endothelium [56]. The plasma concentration of IL-18 has a good correlation with CRP and N-terminal proBrain Natriuretic Peptide [70]. Our recent study demonstrated that IL-18 plays a significant role in the early inflammatory response in patients undergoing open-heart surgery and during the early postoperative period, contributing to increased EG shedding and potentially promoting the development of postoperative complications [71]. In addition, after cardiac surgery, the increased concentration of IL-18 showed a positive correlation with the inflammatory response and acute kidney injury [72]. The activation of the NLRP3 inflammasome is proposed as one of the IL-18-mediated pathways implicated in neurocognitive dysfunction after cardiac surgery [73].

TNF-α exhibited a distinct temporal pattern that is characterized by early elevation during or immediately after CPB, followed by a relatively rapid return toward baseline within 24 h [74,75]. Importantly, pharmacological interventions demonstrated efficacy in attenuating TNF-α responses. Celik et al. showed that preoperative methylprednisolone administration significantly decreased TNF-α levels compared with the placebo in a randomized double-blind trial [75].

The early increase in TNF-α during CPB corresponds with the initial phase of EG shedding, emphasizing its key role as a primary driver of endothelial surface compromise [76,77]. From a molecular standpoint, TNF-α stimulates the production of endothelial heparanase and MMPs, enzymes that degrade the GAG side chains and core proteoglycans involved in anchoring the EG to the endothelial membrane. Heparanase cleaves heparan sulfate chains, the major GAG component of the EG, which provide its structural skeleton, and heparan sulfate degradation leads to the rapid thinning of the EG and destabilization of proteoglycans, thereby facilitating their shedding [78]. Thus, the presence of heparan sulfate fragments and syndecan-1 in the circulation has consistently been used as an indirect marker of EG shedding and has been observed in patients undergoing open-heart surgery. It is noteworthy that heparinase-induced EG degradation not only compromises the integrity of the EG layer but also promotes the release of bound signaling molecules, thereby enhancing the effects of the cytokines involved [79]. In addition, MMPs are an alternative enzymatic pathway through which cytokines such as TNF-α and IL-β induce EG damage, as cytokine activation leads to the transcriptional upregulation of MMPs, which cleave the core proteins of the membrane-bound proteoglycans syndecans and glypicans. This proteolytic cleavage results in the shedding of the remaining GAG chains, while the proteoglycan core proteins are released into the circulation [79,80,81]. This process leads to the loss of the EG architecture, exposing the endothelial membrane to mechanical stress. In the postoperative period, increased MMP activity is correlated with endothelial dysfunction. In addition to membrane-associated EG components, cytokine signaling also regulates the degradation of non-membrane-bound components [33,82]. In addition to enzyme-induced EG degradation, TNF-α also affects the endothelial cytoskeletal structure and the stability of the endothelial intercellular junctions, indirectly compromising EG integrity. Furthermore, TNF-α, along with IL-1β, induces ROS production in the endothelium primarily by activating NADPH oxidases and disrupting mitochondrial electron transport. These processes generate superoxide anions that directly interact with the EG [83].

IL-1β is important in perpetuating cytokine-mediated EG injury through the activation of transcriptional pathways that are involved in maintaining endothelial inflammation. IL-1β, via the NF-κB pathway, regulates the expression of adhesion molecules and pro-inflammatory cytokines, thereby increasing leukocyte–endothelium interactions and contributing to mechanical EG damage [84,85]. IL-1β is also responsible for the induction of oxidative stress through the stimulation of ROS production in the endothelium. ROS directly injure the GAG chains and increase the EG’s susceptibility to proteolytic cleavage [86].

On the other hand, IL-10, as the principal anti-inflammatory cytokine, is consistently elevated after CPB as part of the compensatory anti-inflammatory response that could ameliorate EG shedding [53,57,74]. Giomarelli et al. reported that an increase in IL-10 concentration after CPB was associated with pro-inflammatory injury attenuation, particularly in myocardial and pulmonary tissues [57]. Halter et al. documented IL-10 elevation alongside pro-inflammatory cytokines, suggesting simultaneous activation of both inflammatory and counter-regulatory pathways [53].

**Figure 2:**
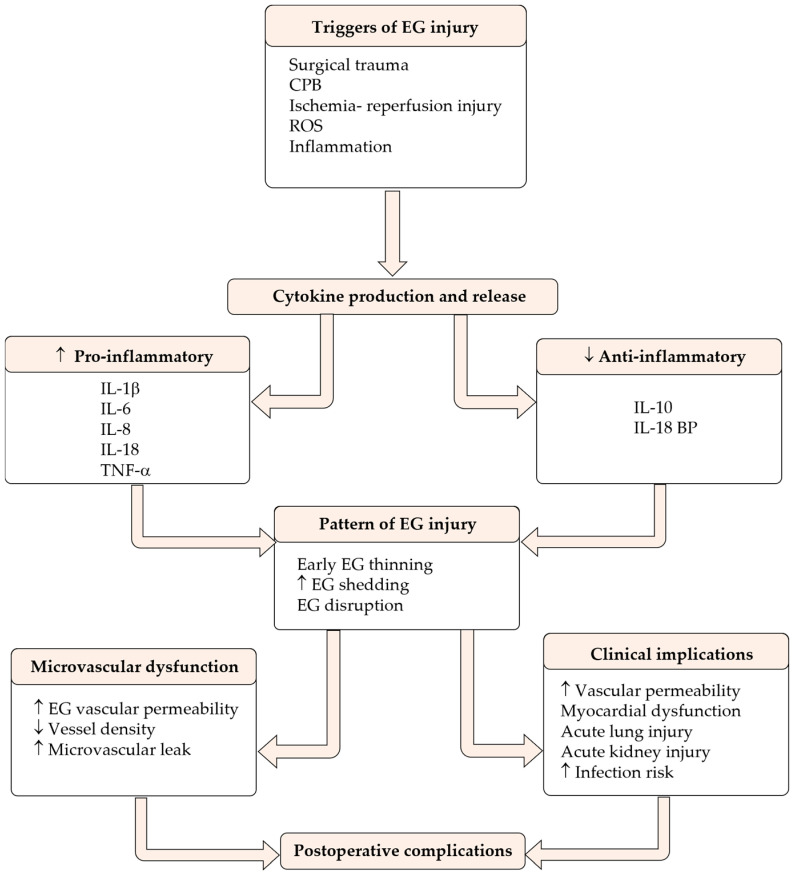
The interplay between cytokines and EG injury during and following open-heart surgery, its clinical implications, and postoperative complications. Surgical trauma and CBP induce ischemia–reperfusion injury, oxidative stress, and inflammation that initiate pro- and anti-inflammatory cytokine production. Increased (↑) production of pro-inflammatory cytokines with decreased (↓) production of anti-inflammatory cytokines promotes EG injury. EG damage contributes to microvascular dysfunction and is associated with adverse clinical implications, ultimately increasing the risk of postoperative complications. Cardiopulmonary bypass (CPB); interleukin (IL); reactive oxygen species (ROS).

Figure 2 summarizes events that precede EG shedding and lead to postoperative complications.

EG degradation by cytokines is further exacerbated by the special hemodynamic and biochemical conditions present during open-heart surgery. For example, altered shear stress during CPB and the oxidative stress caused by ischemia and reperfusion, together with the activation of the endothelium by blood components, all contribute to cytokine-induced EG degradation. In this special environment, the cytokines integrate all forms of mechanical and biochemical stresses and translate them into EG degradation on the endothelial cell surface [37,81].

From a cellular perspective, cytokines activate transcriptional responses in endothelial cells, leading to a pro-inflammatory and pro-degradative response. This response involves the activation of enzymes that cleave the EG, the suppression of protective responses, and the disruption of the cytoskeletal structures that anchor the EG to the cell membrane. All of these events allow the cytokines to induce a change from a barrier-preserving response to a barrier-disrupting response on the endothelial cell surface [78,86]. Thus, the role of cytokine signaling in this process positions it centrally in the mechanisms of postoperative EG injury after cardiac surgery.

## 6. Future Directions and Implications for Targeted Therapeutic Strategies

Patients undergoing open-heart surgery are at a higher risk of developing postoperative complications, partly due to increased circulating concentrations of cytokines that affect EG integrity and function. Therefore, it is important to further investigate the causal relationships between these molecular events and patient outcomes. Measuring dynamic changes in cytokine levels and the EG microstructure may help improve our understanding and prediction of potential postoperative complications, thereby providing an opportunity to determine the optimal time for therapeutic intervention.

Strategies that focus on mitigating the early postoperative cytokine burst may help prevent the initiation of EG shedding. Such strategies may include optimizing CPB circuits, reducing ischemia–reperfusion injury incidence, and modulating surgical stress, all of which have the potential to indirectly preserve EG integrity by reducing excessive proinflammatory cytokine release.

Pharmacological approaches that inhibit cytokine signaling or cytokine receptor activation may further suppress the downstream enzymatic and oxidative mechanisms involved in EG degradation. Immunosuppressive/anti-inflammatory and anti-cytokine-oriented therapies are currently used across a broad spectrum of diseases. By taking accounting of their time-dependent effects during and after cardiac surgery, selective modulation of the cytokines involved in EG degradation (IL-1β, IL-6, IL-18, and TNF-α) or preservation (IL-10) could represent a promising strategy. In the case of IL-1, an additional therapeutic approach may involve inhibition of the inflammasome. Additionally, the development of nanomedicine and the use of nanocarriers may be a useful for reducing systemic immunosuppression. Nanoparticle use has shown promising results in decreasing pro-inflammatory cytokine levels, promoting the synthesis of anti-inflammatory cytokines, and activating anti-inflammatory signaling pathways in cells.

Notably, EG preservation not only involves preventing acute damage but also encompasses promoting EG repair in the postoperative setting. The cytokine-mediated inhibition of endothelial repair mechanisms suggests that therapeutic approaches should extend beyond the intraoperative period and focus on EG restoration. Endothelial homeostasis, NO-mediated signaling, and the supplementation of substrates required for proteoglycan synthesis may facilitate EG repair and improve microvascular function.

In addition to upstream modulation of cytokine effects, inhibiting the downstream effector mechanisms triggered by cytokines is a complementary therapeutic strategy. Moreover, inhibiting EG-degrading enzymes (e.g., heparanase and MMPs), mitigating oxidative stress (e.g., mitochondria-targeted antioxidants), and preserving the endothelial cytoskeletal architecture may help prevent structural damage even in the presence of persistent inflammatory signaling. Early treatment during the peak cytokine response is probably necessary to prevent acute EG damage, whereas delayed therapy may be more effective in promoting EG repair and preventing chronic endothelial dysfunction. Identifying patients with an exaggerated cytokine response or early signs of EG shedding could potentially enable personalized therapy based on circulating biomarkers.

All the proposed targets and therapeutic strategies aim to improve outcomes in cardiac surgery patients by reducing the incidence of postoperative complications that lead to organ dysfunction or systemic inflammatory responses, and the use of therapies directed to specific targets offers the potential to avoid adverse effects at the systemic level. Furthermore, the time-dependent effects of cytokines may allow for improved timing of interventions and their potential integration into surgical and anesthesiologic protocols.

Taken together, these points suggest that cytokine-directed preservation of the EG represents a promising therapeutic strategy in preventing vascular dysfunction during and after open-heart surgery. By interrupting the downstream translation of the inflammatory response into structural endothelial injury, these therapies have the potential to improve outcomes by reducing postoperative complications, enhancing microcirculatory stability, and promoting better postoperative recovery.

## 7. Conclusions

Open-heart surgery induces a complex and highly coordinated systemic inflammatory response that is characterized by the release of pro- and anti-inflammatory cytokines that profoundly influence postoperative outcomes. Cytokines such as IL-1β, IL-6, IL-8, TNF-α, IL-18, and IL-10 act not only as biomarkers of inflammation but also as active mediators linking surgical stress, immune activation, and organ dysfunction. Accumulating evidence demonstrates that cytokine signaling plays a central mechanistic role in EG injury. Early cytokine release initiates enzymatic degradation of EG components by activating heparanase, MMPs, and hyaluronidases, while sustained inflammatory signaling and cytokine-induced oxidative stress impair endothelial repair mechanisms. The resulting loss of EG integrity contributes to the increased vascular permeability, microcirculatory dysfunction, vasoplegia, and multiorgan injury observed after open-heart surgery.

The magnitude and balance of cytokine responses appear to determine the clinical trajectory, emphasizing the importance of inflammatory assessment in open-heart surgery patients. Thus, understanding the interplay between cytokines and EG pathophysiology may provide an opportunity to transition from supportive management toward targeted, mechanism-based therapies aimed at improving organ function and enhancing recovery following open-heart surgery.

## Data Availability

No new data was generated.

## Abbreviations

The following abbreviations are used in this manuscript:

CABG	Coronary Artery Bypass Graft
CPB	Cardiopulmonary Bypass
CRP	C-Reactive Protein
EG	Endothelial Glycocalyx
eNOS	Endothelial Nitric Oxide Synthase
GAGs	Glycosaminoglycans
ICAM	Intercellular Adhesion Molecule
IDF	Incident Dark-Field
IL	Interleukin
IL18-BP	Interleukin-18 Binding Protein
MECC	Minimized Extracorporeal Circulation
MMP	Matrix Metalloproteinase
NETs	Neutrophil Extracellular Traps
NO	Nitric Oxide
PBR	Perfused Boundary Region
PECAM	Platelet Endothelial Cell Adhesion Molecule
ROS	Reactive Oxygen Species
SDF	Sidestream Dark-Field
SIRS	Systemic Inflammatory Response Syndrome
TNF-α	Tumor Necrosis Factor-alpha
VCAM	Vascular Cell Adhesion Molecule

## References

[1] Squiccimarro E., Stasi A., Lorusso R., Paparella D. (2022). Narrative review of the systemic inflammatory reaction to cardiac surgery and cardiopulmonary bypass. Artif. Organs.

[2] Fatehi Hassanabad A., Schoettler F.I., Kent W.D.T., Adams C.A., Holloway D.D., Ali I.S., Novick R.J., Ahsan M.R., McClure R.S., Shanmugam G. (2022). Comprehensive characterization of the postoperative pericardial inflammatory response: Potential implications for clinical outcomes. JTCVS Open.

[3] Khan M.S., Islam M.Y., Ahmed M.U., Bawany F.I., Khan A., Arshad M.H. (2014). On pump coronary artery bypass graft surgery versus off pump coronary artery bypass graft surgery: A review. Glob. J. Health Sci..

[4] Bronicki R.A., Flores S., Loomba R.S., Checchia P.A., Pollak U., Villarreal E.G., Nickerson P., Graham E.M. (2021). Impact of Corticosteroids on Cardiopulmonary Bypass Induced Inflammation in Children: A Meta-Analysis. Ann. Thorac. Surg..

[5] Tan A., Newey C., Falter F. (2022). Pulsatile Perfusion during Cardiopulmonary Bypass: A Literature Review. J. Extra Corpor. Technol..

[6] Squiccimarro E., Labriola C., Malvindi P.G., Margari V., Guida P., Visicchio G., Kounakis G., Favale A., Dambruoso P., Mastrototaro G. (2019). Prevalence and Clinical Impact of Systemic Inflammatory Reaction After Cardiac Surgery. J. Cardiothorac. Vasc. Anesth..

[7] Patterson E.K., Cepinskas G., Fraser D.D. (2022). Endothelial Glycocalyx Degradation in Critical Illness and Injury. Front. Med..

[8] Yamaoka-Tojo M. (2020). Vascular Endothelial Glycocalyx Damage in COVID-19. Int. J. Mol. Sci..

[9] Butler P.J., Bhatnagar A. (2019). Mechanobiology of the abluminal glycocalyx. Biorheology.

[10] Iba T., Levy J.H. (2019). Derangement of the endothelial glycocalyx in sepsis. J. Thromb. Haemost..

[11] Zha D., Fu M., Qian Y. (2022). Vascular Endothelial Glycocalyx Damage and Potential Targeted Therapy in COVID-19. Cells.

[12] Milusev A., Rieben R., Sorvillo N. (2022). The Endothelial Glycocalyx: A Possible Therapeutic Target in Cardiovascular Disorders. Front. Cardiovasc. Med..

[13] Farag E., Esa Y., Chehade N.E.H., Sleiman V.B., Seif J. (2026). Endothelial glycocalyx in perioperative medicine current understanding and future direction. J. Clin. Anesth..

[14] Gomez Toledo A., Golden G.J., Cummings R.D., Malmström J., Esko J.D. (2025). Endothelial Glycocalyx Turnover in Vascular Health and Disease: Rethinking Endothelial Dysfunction. Annu. Rev. Biochem..

[15] Kutuzov N., Flyvbjerg H., Lauritzen M. (2018). Contributions of the glycocalyx, endothelium, and extravascular compartment to the blood-brain barrier. Proc. Natl. Acad. Sci. USA.

[16] Diaz J.A., Gianesini S., Khalil R.A. (2024). Glycocalyx disruption, endothelial dysfunction and vascular remodeling as underlying mechanisms and treatment targets of chronic venous disease. Int. Angiol..

[17] Foote C.A., Soares R.N., Ramirez-Perez F.I., Ghiarone T., Aroor A., Manrique-Acevedo C., Padilla J., Martinez-Lemus L. (2022). Endothelial Glycocalyx. Compr. Physiol..

[18] Jin J., Fang F., Gao W., Chen H., Wen J., Wen X., Chen J. (2021). The Structure and Function of the Glycocalyx and Its Connection with Blood-Brain Barrier. Front. Cell. Neurosci..

[19] Dogné S., Flamion B., Caron N. (2018). Endothelial Glycocalyx as a Shield Against Diabetic Vascular Complications: Involvement of Hyaluronan and Hyaluronidases. Arterioscler. Thromb. Vasc. Biol..

[20] Cosgun Z.C., Fels B., Kusche-Vihrog K. (2020). Nanomechanics of the Endothelial Glycocalyx: From Structure to Function. Am. J. Pathol..

[21] Varki A., Cummings R.D., Esko J.D., Stanley P., Hart G.W., Aebi M., Mohnen D., Kinoshita T., Packer N.H., Prestegard J.H. (2022). Essentials of Glycobiology.

[22] Prydz K. (2015). Determinants of Glycosaminoglycan (GAG) Structure. Biomolecules.

[23] Brouns S.L.N., Provenzale I., van Geffen J.P., van der Meijden P.E.J., Heemskerk J.W.M. (2020). Localized endothelial-based control of platelet aggregation and coagulation under flow: A proof-of-principle vessel-on-a-chip study. J. Thromb. Haemost..

[24] Villalba N., Baby S., Yuan S.Y. (2021). The Endothelial Glycocalyx as a Double-Edged Sword in Microvascular Homeostasis and Pathogenesis. Front. Cell Dev. Biol..

[25] Gaudette S., Hughes D., Boller M. (2020). The endothelial glycocalyx: Structure and function in health and critical illness. J. Vet. Emerg. Crit. Care.

[26] Qu J., Cheng Y., Wu W., Yuan L., Liu X. (2021). Glycocalyx Impairment in Vascular Disease: Focus on Inflammation. Front. Cell Dev. Biol..

[27] Chevalier L., Selim J., Castro C., Cuvilly F., Baste J.M., Richard V., Pareige P., Bellien J. (2022). Combined Electron Microscopy Approaches for Arterial Glycocalyx Visualization. Front. Cardiovasc. Med..

[28] Di X., Gao X., Peng L., Ai J., Jin X., Qi S., Li H., Wang K., Luo D. (2023). Cellular mechanotransduction in health and diseases: From molecular mechanism to therapeutic targets. Sig. Transduct. Target Ther..

[29] Hu Z., Cano I., D’Amore P.A. (2021). Update on the Role of the Endothelial Glycocalyx in Angiogenesis and Vascular Inflammation. Front. Cell Dev. Biol..

[30] Ushiyama A., Kataoka H., Iijima T. (2016). Glycocalyx and its involvement in clinical pathophysiologies. J. Intensive Care.

[31] Wiesinger A., Peters W., Chappell D., Kentrup D., Reuter S., Pavenstädt H., Oberleithner H., Kümpers P. (2013). Nanomechanics of the Endothelial Glycocalyx in Experimental Sepsis. PLoS ONE.

[32] Schött U., Solomon C., Friès D., Bentzer P. (2016). The glycocalyx and its disruption, protection and regeneration: A narrative review. Scand. J. Trauma Resusc. Emerg. Med..

[33] Kršek A., Batičić L., Ćurko-Cofek B., Batinac T., Laškarin G., Miletić-Gršković S., Sotošek V. (2024). Insights into the Molecular Mechanism of Endothelial Glycocalyx Dysfunction during Heart Surgery. Curr. Issues Mol. Biol..

[34] Ferreira G., Taylor A., Mensah S.A. (2024). Deciphering the triad of endothelial glycocalyx, von Willebrand Factor, and P-selectin in inflammation-induced coagulation. Front. Cell Dev. Biol..

[35] van der Poll T., Parker R.I. (2020). Platelet Activation and Endothelial Cell Dysfunction. Crit. Care.

[36] Uchimido R., Schmidt E.P., Shapiro N.I. (2019). The glycocalyx: A novel diagnostic and therapeutic target in sepsis. Crit. Care.

[37] Sullivan R.C., Rockstrom M.D., Schmidt E.P., Hippensteel J.A. (2021). Endothelial glycocalyx degradation during sepsis: Causes and consequences. Matrix Biol. Plus.

[38] Ćurko-Cofek B., Jenko M., Taleska Stupica G., Batičić L., Krsek A., Batinac T., Ljubačev A., Zdravković M., Knežević D., Šoštarič M. (2024). The crucial triad: Endothelial glycocalyx, oxidative stress, and inflammation in cardiac surgery. Int. J. Mol. Sci..

[39] Knežević D., Ćurko-Cofek B., Batinac T., Laškarin G., Rakić M., Šoštarič M., Zdravković M., Šustić A., Sotošek V., Batičić L. (2023). Endothelial Dysfunction in Patients Undergoing Cardiac Surgery: A Narrative Review and Clinical Implications. J. Cardiovasc. Dev. Dis..

[40] Robich M.P., Ryzhov S., Kacer D., Palmeri M., Peterson S.M., Quinn R.D., Carter D., Sheppard F., Hayes T., Sawyer D.B. (2020). Prolonged cardiopulmonary bypass is associated with endothelial glycocalyx degradation. J. Surg. Res..

[41] Reitsma S., Slaaf D.-W., Vink H., van Zandvoort M.A.M.J., oude Egbrink M.G.A. (2007). The endothelial glycocalyx: Composition, functions, and visualization. Pflügers Arch..

[42] Li S., Nordick K.V., Murrieta-Álvarez I., Kirby R.P., Bhattacharya R., Garcia I., Hochman-Mendez C., Rosengart T.K., Liao K.K., Mondal N.K. (2025). Prolonged cardiopulmonary bypass time-induced endothelial dysfunction via glypican-1 shedding, inflammation, and matrix metalloproteinase 9 in patients undergoing cardiac surgery. Biomedicines.

[43] Bol M.E., Huckriede J.B., van de Pas K.G.H., Delhaas T., Lorusso R., Nicolaes G.A.F., Sels J.E.M., van de Poll M.C.G. (2022). Multimodal measurement of glycocalyx degradation during coronary artery bypass grafting. Front. Med..

[44] Henry C.B.S., Duling B.R. (1999). Permeation of the luminal capillary glycocalyx is determined by hyaluronan. Am. J. Physiol. Heart Circ. Physiol..

[45] Aldecoa C., Llau J.V., Nuvials X., Artigas A. (2020). Role of albumin in the preservation of endothelial glycocalyx integrity. Ann. Intensive Care.

[46] Kim H.B., Soh S., Kwak Y.L., Bae J.C., Kang S.H., Song J.W. (2020). High preoperative serum syndecan-1 and severe acute kidney injury after valvular heart surgery. J. Clin. Med..

[47] de Melo Bezerra Cavalcante C.T., Castelo Branco K.M., Pinto Júnior V.C., Meneses G.C., de Oliveira Neves F.M., de Souza N.M., Penaforte K.L., Martins A.M., Libório A.B. (2016). Syndecan-1 improves severe acute kidney injury prediction after pediatric cardiac surgery. J. Thorac. Cardiovasc. Surg..

[48] Xu J., Jiang W., Li Y., Li H., Geng X., Chen X., Hu J., Shen B., Wang Y., Fang Y. (2021). Association Between Syndecan-1, Fluid Overload, and Progressive Acute Kidney Injury After Adult Cardiac Surgery. Front. Med..

[49] Budiwardhana N., Murni I.K., Marwali E.M., Busro P.W., Rizkia F.I., Soelaeman M.F., Widyastuti Y. (2025). Correlation between Syndecan-1 in Inter Category of RACHS-1 Score and Immediate Clinical Outcomes. Congenit. Heart Dis..

[50] Diaz D.M., Orton E.C., de Rezende M.L., Zersen K., Guillaumin J. (2023). Assessment of microcirculation variables and endothelial glycocalyx using sidestream dark field videomicroscopy in anesthetized dogs undergoing cardiopulmonary bypass. Front. Vet. Sci..

[51] Cusack R., Leone M., Rodriguez A.H., Martin-Loeches I. (2022). Endothelial Damage and the Microcirculation in Critical Illness. Biomedicines.

[52] Tønnesen E., Christensen V.B., Toft P. (1996). The role of cytokines in cardiac surgery. Int. J. Cardiol..

[53] Halter J.M., Steinberg J., Fink G., Lutz C., Picone A., Maybury R., Fedors N., DiRocco J., Lee H.M., Nieman G. (2005). Evidence of systemic cytokine release in patients undergoing cardiopulmonary bypass. J. Extra Corpor. Technol..

[54] Laffey J.G., Boylan J.F., Cheng D.C. (2002). The systemic inflammatory response to cardiac surgery: Implications for the anesthesiologist. Anesthesiology.

[55] Holmes J.H., Connolly N.C., Paull D.L., Hill M.E., Guyton S.W., Ziegler S.F., Hall R.A. (2002). Magnitude of the inflammatory response to cardiopulmonary bypass and its relation to adverse clinical outcomes. Inflamm. Res..

[56] Venkatachalam K., Prabhu S.D., Reddy V.S., Boylston W.H., Valente A.J., Chandrasekar B. (2009). Neutralization of interleukin-18 ameliorates ischemia/reperfusion-induced myocardial injury. J. Biol. Chem..

[57] Giomarelli P., Scolletta S., Borrelli E., Biagioli B. (2003). Myocardial and lung injury after cardiopulmonary bypass: Role of interleukin (IL)-10. Ann. Thorac. Surg..

[58] Habes Q.L.M., Kant N., Beunders R., van Groenendael R., Gerretsen J., Kox M., Pickkers P. (2023). Relationships between systemic inflammation, intestinal damage and postoperative organ dysfunction in adults undergoing low-risk cardiac surgery. Heart Lung Circ..

[59] Martínez-Comendador J., Alvarez J.R., Mosquera I., Sierra J., Adrio B., Carro J.G., Fernández A., Bengochea J. (2009). Preoperative statin treatment reduces systemic inflammatory response and myocardial damage in cardiac surgery. Eur. J. Cardiothorac. Surg..

[60] Drost C.C., Rovas A., Osiaevi I., Schughart K., Lukasz A., Linke W.A., Pavenstädt H., Kümpers P. (2024). Interleukin-6 drives endothelial glycocalyx damage in COVID-19 and bacterial sepsis. Angiogenesis.

[61] Yu A., Amrute J.M., Eghtesady P. (2025). Review of Interleukin-6 and Cardiopulmonary Bypass-Related End-Organ Injury Along with the Potential for Mitigation with Tocilizumab. World J. Pediatr. Congenit. Heart Surg..

[62] Velusamy P., Buckley D.J., Greaney J.L., Case A.J., Fadel P.J., Trott D.W. (2025). IL-6 induces mitochondrial ROS production and blunts NO bioavailability in human aortic endothelial cells. Am. J. Physiol. Regul. Integr. Comp. Physiol..

[63] Cuevas-Budhart M.A., Sánchez-Garre M., Sánchez-Bermúdez A., Sobrino-Rodríguez A., Arniella-Blanco M.M., Renghea A., Crespo-Cañizares A., Cavero-Redondo I., Gallardo J.M., Gómez del Pulgar M. (2025). Oxidative Stress and Postoperative Outcomes: An Umbrella Review of Systematic Reviews and Meta-Analyses. Antioxidants.

[64] Yan R., Zhang X., Xu W., Li J., Sun Y., Cui S., Xu R., Li W., Jiao L., Wang T. (2024). ROS-Induced Endothelial Dysfunction in the Pathogenesis of Atherosclerosis. Aging Dis..

[65] Roca F.J., Whitworth L.J., Prag H.A., Murphy M.P., Ramakrishnan L. (2022). Tumor necrosis factor induces pathogenic mitochondrial ROS in tuberculosis through reverse electron transport. Science.

[66] Saelim K., Ruangnapa K., Jarutach J., Kaukinen S., Honkonen E.L., Metsänoja R., Tarkka M. (2025). Cytokine profile of post-cardiopulmonary bypass in children. Clin. Exp. Pediatr..

[67] Wei M., Kuukasjarvi P., Laurikka J., Kaukinen S., Honkonen E., Metsänoja R., Tarkka M. (2003). Relation of cytokines to vasodilation after coronary artery bypass grafting. World J. Surg..

[68] Elkhatib W.Y., Saunders H., Helgeson S.A., Moss J.E. (2021). The use of an interleukin-6 inhibitor in vasoplegic shock from severe systemic inflammatory response syndrome: A case report. Indian J. Crit. Care Med..

[69] Derler R., Gesslbauer B., Weber C., Strutzmann E., Miller I., Kungl A. (2017). Glycosaminoglycan-Mediated Downstream Signaling of CXCL8 Binding to Endothelial Cells. Int. J. Mol. Sci..

[70] Kawasaki D., Tsujino T., Morimoto S., Masai M., Masutani M., Ohyanagi M., Kashiwamura S., Okamura H., Masuyama T. (2005). Plasma interleukin-18 concentration: A novel marker of myocardial ischemia rather than necrosis in humans. Coron. Artery Dis..

[71] Knežević D., Batičić L., Ćurko-Cofek B., Batinac T., Ljubačev A., Valenčić Seršić L., Laškarin G., Zdravković M., Šoštarič M., Sotošek V. (2025). The Effect of Coronary Artery Bypass Surgery on Interleukin-18 Concentration and Biomarkers Related to Vascular Endothelial Glycocalyx Degradation. Int. J. Mol. Sci..

[72] Coca S.G., Nadkarni G.N., Garg A.X., Koyner J., Thiessen-Philbrook H., McArthur E., Shlipak M.G., Parikh C.R., TRIBE-AKI Consortium (2016). First post-operative urinary kidney injury biomarkers and association with the duration of AKI in the TRIBE-AKI cohort. PLoS ONE.

[73] Ma G., Sun P., Chen Y., Jiang X., Zhang C., Qu B., Meng X. (2022). NLRP3 inflammasome activation contributes to the cognitive decline after cardiac surgery. Front. Surg..

[74] Roth-Isigkeit A., Borstel T.V., Seyfarth M., Schmucker P. (1999). Perioperative serum levels of tumour-necrosis-factor alpha (TNF-α), IL-1β, IL-6, IL-10 and soluble IL-2 receptor in patients undergoing cardiac surgery with cardiopulmonary bypass. Clin. Exp. Immunol..

[75] Celik J.B., Gormus N., Okesli S., Gormus Z.I., Solak H. (2004). Methylprednisolone prevents inflammatory reaction occurring during cardiopulmonary bypass: Effects on TNF-α, IL-6, IL-8, IL-10. Perfusion.

[76] Qu R., Du W., Li S., Li W., Wei G., Chen Z., Gao H., Shi S., Zou L., Li H. (2024). Destruction of vascular endothelial glycocalyx during formation of pre-metastatic niches. Heliyon.

[77] Hohn A., Malewicz-Oeck N.M., Buchwald D., Annecke T., Zahn P.K., Baumann A. (2024). REmoval of cytokines during CArdiac surgery (RECCAS): A randomised controlled trial. Crit. Care.

[78] Yung S., Chan T.M. (2023). Endothelial cell activation and glycocalyx shedding–potential as biomarkers in patients with lupus nephritis. Front. Immunol..

[79] Inoda A., Suzuki K., Tomita H., Okada H. (2025). Glycocalyx shedding as a clinical biomarker in critical illness. Exp. Mol. Pathol..

[80] Li H., Wen H., Liu J., Luo X., Pei B., Ge P., Sun Z., Liu J., Wang J., Chen H. (2025). The glycocalyx: A key target for treatment of severe acute pancreatitis-associated multiple organ dysfunction syndrome. Hum. Cell.

[81] Wang J., Ma L., Fang Y., Ye T., Li H., Lan P. (2025). Factors influencing glycocalyx degradation: A narrative review. Front. Immunol..

[82] Drinhaus H., Mallmann C., Cleff C., Neumann T., Daniels C., Bruns C.J., Steinbicker A.U., Schröder W., Annecke T. (2025). Glycocalyx-Shedding and Inflammatory Reactions Occur Yet Do Not Predict Complications Resulting from an Esophagectomy in an Accelerated Recovery After Surgery Program. J. Clin. Med..

[83] Kipcke J.P., Odenthal-Schnittler M., Aldirawi M., Franz J., Bojovic V., Seebach J., Schnittler H. (2025). TNF-α induces VE-cadherin-dependent gap/JAIL cycling through an intermediate state essential for neutrophil transmigration. Front. Immunol..

[84] Mao H., Zhao X., Sun S.C. (2025). NF-κB in inflammation and cancer. Cell Mol. Immunol..

[85] Kihara T., Toriuchi K., Aoki H., Kakita H., Yamada Y., Aoyama M. (2021). Interleukin-1β enhances cell adhesion in human endothelial cells via microRNA-1914-5p suppression. Biochem. Biophys. Rep..

[86] Xia T., Yu J., Du M., Chen X., Wang C., Li R. (2025). Vascular endothelial cell injury: Causes, molecular mechanisms, and treatments. MedComm.

